# Artificial intelligence as a second reader for pneumothorax detection: redirecting rather than reducing chest CT use

**DOI:** 10.1186/s12873-026-01673-x

**Published:** 2026-07-02

**Authors:** Muhammed Fatih Cırıl, Ferhat Yıldız, Mustafa Akarca

**Affiliations:** Department of Emergency Medicine, Mardin Training and Research Hospital, Artuklu, Mardin, Türkiye

**Keywords:** Pneumothorax, Artificial intelligence, Deep learning, Chest radiography, Computed tomography, Emergency department, Clinical decision-making, Diagnostic imaging

## Abstract

**Background:**

Pneumothorax is a finding that is frequently missed in the emergency department, particularly in cases with subtle radiographic features, and can rapidly progress to tension pneumothorax when overlooked on the initial chest radiograph. Although artificial intelligence (AI)–assisted chest radiograph interpretation has been shown to improve diagnostic accuracy, its effect on the actual decision behaviour of emergency physicians — particularly on chest computed tomography (CT) ordering — remains insufficiently studied. This study evaluated the impact of AI-assisted chest radiograph interpretation on emergency physicians’ pneumothorax diagnostic decisions and chest CT ordering behaviour, with a particular focus on the capacity of the AI alert to function as a second reader and capture diagnoses initially missed by the physician.

**Methods:**

This was a nationwide, online, cross-sectional simulation study conducted between 1 and 15 April 2026. Emergency physicians practising in Türkiye were asked to interpret 20 standardised posteroanterior chest radiographs presented in a randomised order (10 pneumothorax-positive [5 overt and 5 subtle] and 10 pneumothorax-negative cases, all confirmed by chest CT). For each case, participants first indicated whether they suspected pneumothorax on the unannotated radiograph, and then — after being shown the same image annotated by a CE-certified, deep learning–based chest radiograph interpretation system — indicated whether they would order a chest CT. The primary endpoint was the paired pre-AI versus post-AI change in simulated CT-ordering intent, derived from a predefined survey coding scheme and analysed using the McNemar exact test. As the pre- and post-AI items do not capture CT-ordering intent identically, this endpoint reflects simulated imaging intent rather than observed CT-ordering behaviour.

**Results:**

A total of 231 physicians (51.5% general practitioners, 25.5% emergency medicine residents, 22.9% emergency medicine specialists) completed the survey, yielding 4,620 physician–case response pairs. Simulated CT-ordering intent rose from 8.1% before AI exposure to 26.7% after AI exposure (absolute difference + 18.6% points; *p* < 0.001). The increase was concentrated in pneumothorax-positive cases (+ 31.6 points; *p* < 0.001) and remained limited in pneumothorax-negative cases (+ 5.7 points; *p* < 0.001). In subtle pneumothorax cases, the integrated diagnostic capture rate rose from 54.8% to 85.0% (+ 30.2 points); among the 339 cases in which physicians had initially answered “no pneumothorax,” 211 (62.2%) were converted into a CT order after the AI alert. The effect was statistically significant across all professional titles and seniority strata (*p* < 0.001 for all). The mean perceived usability score of the system was 8.1 out of 10 (interquartile range 7–10).

**Conclusions:**

In this simulation, AI-assisted chest radiograph interpretation did not reduce simulated CT-ordering intent; rather, the increase in intent was concentrated in true positive cases, with only a limited effect on negative cases. These findings are hypothesis-generating and require prospective confirmation with real CT-ordering data. The principal clinical value of the system lies in capturing pneumothorax diagnoses initially missed by emergency physicians, supporting its positioning as a second reader rather than as a CT-reduction tool.

**Trial registration:**

Not applicable.

**Supplementary Information:**

The online version contains supplementary material available at https://doi.org/10.1186/s12873-026-01673-x.

## Background

Pneumothorax is among the uncommon presentations in which a small visual cue may translate into a disproportionately large mortality risk: once missed on the chest radiograph [1], the same patient can rapidly progress to tension pneumothorax after discharge from the emergency department or after initiation of positive-pressure ventilation, in whom haemodynamic collapse develops far more frequently [2]. This asymmetry — the cost of the error far exceeding the difficulty of the diagnosis — turns pneumothorax screening, and with it the deceptively “simple” posteroanterior chest radiograph, into one of the most consequential decisions made in the emergency department. Screening sensitivity hinges on whether the reader notices a one-millimetre pleural line on the film; that single observation determines whether the patient is discharged, admitted, or taken for intervention in the next hour.

The chest radiograph is the first step in this decision: inexpensive, rapid, and immediately available in virtually every emergency department. In cases of overt large pneumothorax the diagnosis is relatively straightforward and is captured with high sensitivity even by less experienced readers, whereas sensitivity falls markedly in subtle cases [3, 4]. The difficulty begins in subtle cases. In apical, small-volume pneumothoraces, and in those obscured by subcutaneous emphysema (air within the soft tissues of the chest wall) or rib shadows, reader experience becomes the principal determinant of sensitivity; in a 563-radiograph emergency department cohort, the fraction of pneumothorax cases flagged by board-certified radiologists but reached by none of the readers in the non-radiology resident group (a group consensus score of zero) approached 50% [3]. For this reason, computed tomography retains its position as the gold standard for pneumothorax in both trauma and non-trauma populations [1]. CT’s status as the reference standard, however, does not justify a low threshold for CT ordering in practice; radiation exposure, cost, prolonged patient throughput, and the impact on emergency department crowding force a balancing calculation on every physician for every case.

This balance becomes even more demanding in the Turkish emergency department setting. Initial interpretation of the chest radiograph is performed in most centres by the emergency physician; on night shifts or when radiology staffing is limited, a general practitioner or an emergency medicine resident may be the sole decision-maker. In a context where structural 24/7 radiologist access is constrained [3], the probability of missing a subtle pneumothorax rises systematically — the problem is less attributable to the individual physician’s attention than to the system itself.

AI-assisted chest radiograph interpretation is one of the leading technological developments positioned to address this structural gap. Recent multicentre MRMC studies have reported that AI assistance produces statistically significant improvements in pneumothorax diagnostic accuracy [4–7], with stronger effects in junior readers and in subtle cases [6–8], and that AI can deliver more consistent performance than physicians in such cases [8, 9]. In a recent emergency-department study on spontaneous pneumothorax from the same country, a vision-enabled model estimated pneumothorax depth in close agreement with radiologist measurements (intraclass correlation coefficient 0.893) and predicted initial management with 90.1% accuracy and an area under the curve of 0.90 [10]. The role of generative AI models in emergency department chest radiograph interpretation has also been examined: AI-generated reports have been shown to achieve quality scores comparable to those of radiologists and to outperform teleradiology reports [11]. The capacity of clinical decision support systems to alter CT ordering behaviour in the emergency department has likewise been investigated, with some centres reporting significant reductions [12] and others reporting no clear effect [13]. Systematic reviews of machine-learning–based decision support tools, however, caution that the existing evidence base on whether such tools actually improve clinician performance remains methodologically limited [14].

Three notable gaps emerge within this body of work. First, a substantial proportion of the studies have been performed with radiologists or radiology residents, whereas in many countries — Türkiye among them — the first reader of the chest radiograph is the emergency physician rather than the radiologist. How emergency physicians respond to an AI alert remains comparatively understudied. Second, most of the available evidence measures diagnostic accuracy; the number of studies directly measuring whether and in which case types an AI alert redirects the physician towards a concrete decision — for example, ordering a chest CT — remains small [5, 15]. Third, when these two outcomes (diagnostic accuracy and advanced imaging request) are examined simultaneously in the same participant pool, the actual source of the clinical value of AI assistance becomes visible: is it the capture of findings initially overlooked and their conversion into a CT order, or is it the confirmation of decisions that were already correct? This question requires reframing AI not as a stand-alone diagnostic tool but as a “second reader” — an additional pair of eyes that joins the physician precisely in the cases that are most likely to be missed.

The aim of this study was to evaluate, in parallel, the pneumothorax diagnostic decisions and chest CT ordering behaviour of emergency physicians across Türkiye on standardised chest radiograph cases before and after AI-assisted interpretation, and to characterise the direction, magnitude, and subgroup distribution of the AI alert’s influence on physician decisions as a second reader — with particular emphasis on the extent to which initially missed diagnoses are recovered.

## Methods

### Study design and ethical statement

This was an online, survey-based, cross-sectional simulation study designed to assess the pneumothorax diagnostic decisions and chest CT ordering behaviour of emergency physicians practising in Türkiye before and after AI-assisted chest radiograph interpretation. Data collection and statistical analysis were planned and reported in accordance with the STROBE-Cross Sectional recommendations.

The study protocol was approved by the Scientific Research Ethics Committee of Mardin Training and Research Hospital on 27 March 2026 under protocol number 2026/03 − 01 (3rd meeting, decision number 01). All stages of the study were conducted in accordance with the current version of the Declaration of Helsinki. The introductory page of the online survey contained information regarding the study aim, the voluntary nature of participation, the non-collection of personal and electronic mail data, and the use of the data for academic purposes only; participants who proceeded with the survey were considered to have read and acknowledged this information. All patient-identifying details on the chest radiographs were masked, and the images were anonymised before inclusion.

### Study setting and participants

The survey was developed at the emergency department of Mardin Training and Research Hospital and distributed nationally online. The participant pool consisted of general practitioners, emergency medicine residents, and emergency medicine specialists. Participation was voluntary; physicians invited to and accepting the survey were included. Data were collected between 1 and 15 April 2026.

The survey was prepared on Google Forms (Google LLC, Mountain View, CA, USA) and delivered online to emergency physicians practising in Türkiye. Each participant was permitted to respond only once; duplicate and incomplete responses were excluded from the analysis.

#### Inclusion criteria

General practitioners, emergency medicine residents, and emergency medicine specialists actively working in an emergency department in Türkiye during the survey period and providing complete responses.

#### Exclusion criteria

Participants who left at least one mandatory item in the demographic or case sections unanswered, or who provided conflicting multiple answers.

### Case selection and reference diagnosis

The 20 standardised chest radiographs used in the survey were retrospectively selected from the Picture Archiving and Communication System (PACS) of Mardin Training and Research Hospital from the records of adult patients who had presented to the emergency department within the six months preceding the data collection period. All radiographs were obtained in the posteroanterior (PA) projection. Images were acquired on fixed radiographic equipment in the radiology department, in the erect position, using a standard posteroanterior technique; no bedside or portable (mobile) radiographs were included. Radiographs were exported from PACS without additional cropping or post-processing. In this setting, radiographers acquire the image but do not provide a diagnostic interpretation, so the emergency physician is the first diagnostic reader of the chest radiograph.

Cases were divided into two main pools: 10 pneumothorax-positive and 10 pneumothorax-negative. This 10:10 ratio represents a deliberate, controlled enrichment intended to provide adequate exposure to both case types within a simulation; it does not reflect the prevalence of pneumothorax among routine emergency-department chest radiographs, and the findings should therefore be read as estimates under a selected case-mix simulation rather than under routine prevalence conditions. The pneumothorax-positive pool was subsequently subdivided according to visual volume assessment into overt pneumothorax (5 cases) and subtle pneumothorax (5 cases). The overt/subtle distinction was based on a visual volume assessment performed by the study authors (using whether hemithorax involvement roughly exceeded 50%), without independent radiologist adjudication or formal volumetric measurement (e.g. Light index, Collins method); the subtle group preferentially included apical, small-volume cases. This classification approach is acknowledged as a limitation.

#### Reference diagnosis

The true diagnosis for all cases (both positive and negative) was confirmed by chest computed tomography obtained during the same clinical presentation. Only patients in whom a chest CT examination was available and the diagnosis was substantiated by the CT report were included. This approach ensured that the reference classification of the study was anchored to a gold standard, guaranteeing that PTX-positive cases were truly positive and PTX-negative cases truly negative. Each radiograph was accompanied by a brief clinical context label (age + main symptoms; e.g. “30 years, female, chest pain, cough”) in order to reflect actual patient flow.

Patient names, protocol numbers, and other identifying fields were visually masked during selection.

### Artificial intelligence support system

The AI support system used in the study was hChestXR (Hevi AI, Istanbul, Türkiye), software that has been in routine clinical use in the PACS of the emergency department of Mardin Training and Research Hospital for approximately 1.5 years. The software is a deep learning–based image analysis tool developed to detect eight major pathologies on chest radiographs (including pneumothorax) and carries MDR-CE Class IIa certification. The system automatically analyses each radiograph at the moment it arrives in the PACS and displays, on the same screen, a coloured bounding box delineating the anatomical region together with a numerical confidence score on a 1–100 scale. Scores of 50 and above are highlighted as high-probability findings. During the survey, the “post-AI” image for each case was presented using the original annotated screen output produced by hChestXR; in pneumothorax-positive cases a purple bounding box and the label “Pneumothorax” appear together with the numerical score, whereas no annotation is shown in pneumothorax-negative cases. Because the image set was drawn exclusively from CT-confirmed cases and no radiograph on which the AI produced an incorrect output was included, every AI output in the study was concordant with the CT-confirmed reference diagnosis (all positive cases AI-positive, all negative cases AI-negative). The study therefore evaluates physician responses to correct AI output and does not test behaviour under AI error conditions.

None of the authors had any role in the development or commercialisation of the software, and no financial support was received from the manufacturer for this study.

### Survey flow and endpoints

**The first section** collected demographic data: professional title (general practitioner, emergency medicine resident, emergency medicine specialist, other) and total time in practice (recent graduate, less than 1 year, 1–3 years, 4–6 years, 6–10 years, 10 + years).

**The second section** comprised the sequential simulation of 20 standardised cases. For each case, the participant was first presented with the unannotated (pre-AI) chest radiograph and the brief clinical context, and asked, “Do you consider that pneumothorax is present on this chest radiograph?” (question a; three options: “Yes”; “No”; “I am not sure, I would order further imaging [chest CT]”). After this initial response on the same screen, the same case was presented with the hChestXR annotation, and the participant was asked, “After seeing the AI annotation on this patient’s radiograph, would you order a chest CT for this patient?” (question b; four options: “Yes, I would — I want a CT despite the AI”; “No, I would not — the AI made me drop the CT”; “I was not going to anyway and still would not — the AI reinforced my decision”; “I was going to anyway and still would — the AI did not change my decision”). The 20 cases were presented in randomised order to each participant; overt pneumothorax, subtle pneumothorax, and pneumothorax-negative cases were interleaved. All participants saw the same clinical context labels.

**The third section** consisted of two general opinion items at the end of the survey. The first, “Do you think AI-assisted chest radiograph interpretation can reduce the rate of unnecessary chest CT scans in the emergency department?“, was rated on a 5-point Likert scale (definitely yes → definitely no). The second, “What do you think of the usability of such an AI support system in your daily clinical practice?“, was rated on a numerical scale from 1 to 10 (1: not useful at all, 10: indispensable support).

**Endpoints**: The primary endpoint was the pre-AI versus post-AI change in simulated chest CT-ordering intent measured at the case level, derived from the predefined coding scheme described below. Secondary endpoints were: (i) the physician’s pneumothorax diagnostic accuracy (sensitivity, specificity, overall accuracy; with overt/subtle subgroup analyses); (ii) the integrated diagnostic capture rate after the AI alert (a diagnosis was considered “captured” if the physician had either answered “pneumothorax present” initially or ordered a CT after the AI alert); (iii) the categorisation of changes in CT ordering behaviour (“moved to CT,” “dropped CT,” “persisted with CT,” “already not ordering”); and (iv) physicians’ perceptions of the usability and utility of the AI system.

### Variable coding

For the primary endpoint analysis, questions a and b were transformed into two binary variables. The **pre-AI CT ordering** variable was assigned 1 if a = “I am not sure, I would order a CT,” and 0 if a = “Yes” or “No.” The **post-AI CT ordering** variable was assigned 1 if b = “I want a CT despite the AI” or “I was going to anyway and still would,” and 0 if b = “The AI made me drop the CT” or “I was not going to anyway and still would not.” This approach allowed the physician’s final clinical decision (to order or not to order a CT) to be modelled as a binary outcome.

**Pneumothorax interpretation (pre-AI)**: a = “Yes” → “PTX present,” a = “No” → “PTX absent,” a = “Not sure” → “indeterminate.” Two approaches were used in diagnostic accuracy calculations: in the base calculation, “indeterminate” responses were excluded from the analysis, whereas in the strict (conservative) calculation, “indeterminate” responses were considered as misclassifications.

**Handling of inconsistent responses**: As a natural consequence of the survey design, certain a/b combinations could yield logically conflicting outcomes (e.g. a physician answering “PTX present” in question a and “The AI made me drop the CT” in question b). These responses (1,395 of the total 4,620, or 30.2%) were not excluded from the analysis; question b was taken as the reference in the CT ordering analysis, as it reflects the physician’s final clinical stance. This approach is examined in detail in the Discussion section together with automation bias.

### Statistical analysis

Categorical variables are presented as n (%), normally distributed continuous variables as mean ± standard deviation, and non-normally distributed continuous variables as median [interquartile range, IQR]. Normality was assessed using the Shapiro–Wilk test.

For the primary endpoint (the difference between pre-AI and post-AI simulated CT-ordering intent), the McNemar exact test (two-sided binomial) was applied to the paired binary data. Effect sizes are reported as absolute percentage-point changes. Subgroup analyses were performed separately for professional title (general practitioner, emergency medicine resident, emergency medicine specialist), seniority (recent graduate, 1–3 years, 4–10 years, 10 + years), and case type (pneumothorax-positive/negative, overt/subtle). Title × case type cross-analyses were likewise evaluated with the McNemar exact test.

Physicians’ diagnostic accuracy for pneumothorax (sensitivity, specificity, and overall accuracy) was calculated with 95% confidence intervals. The integrated capture rate was defined as the proportion of pneumothorax-positive cases in which the physician had either answered “PTX present” at baseline or ordered a CT after the AI alert.

No correction for multiple testing was applied; the hypothesis-driven primary endpoint of the study was a single pre-specified comparison (overall simulated CT-ordering intent pre-AI vs. post-AI), and the subgroup analyses are reported as exploratory. Under a Bonferroni-adjusted threshold (α = 0.05/8 = 0.00625), seven of the eight comparisons in Table 8 retained significance; only the emergency medicine specialist × pneumothorax-negative comparison (*p* = 0.022) fell above this threshold. All analyses were performed in Python version 3 (pandas, scipy.stats libraries). The statistical significance threshold was set at *p* < 0.05; p values were derived from McNemar exact test results.

The sample size was not determined a priori by formal power calculation, given the cross-sectional, paired design and the multiple planned subgroup analyses; the aim was to recruit the largest feasible participant pool. At the end of the distribution period, 231 participants were obtained, corresponding to 4,620 physician–case response pairs as each physician evaluated 20 cases. A post-hoc power analysis using the observed number of discordant pairs (*n* = 1,071; p21 = 0.209; p12 = 0.023) and a total of *N* = 4,620 response pairs was performed with the Connor (1987) method using the scipy.stats library; the detected power at a two-sided α = 0.05 was > 0.999. The sample size required to detect a more modest absolute difference of 5% points at 80% power would be approximately 730 response pairs, well below the available sample.

## Results

**Fig. 1:**
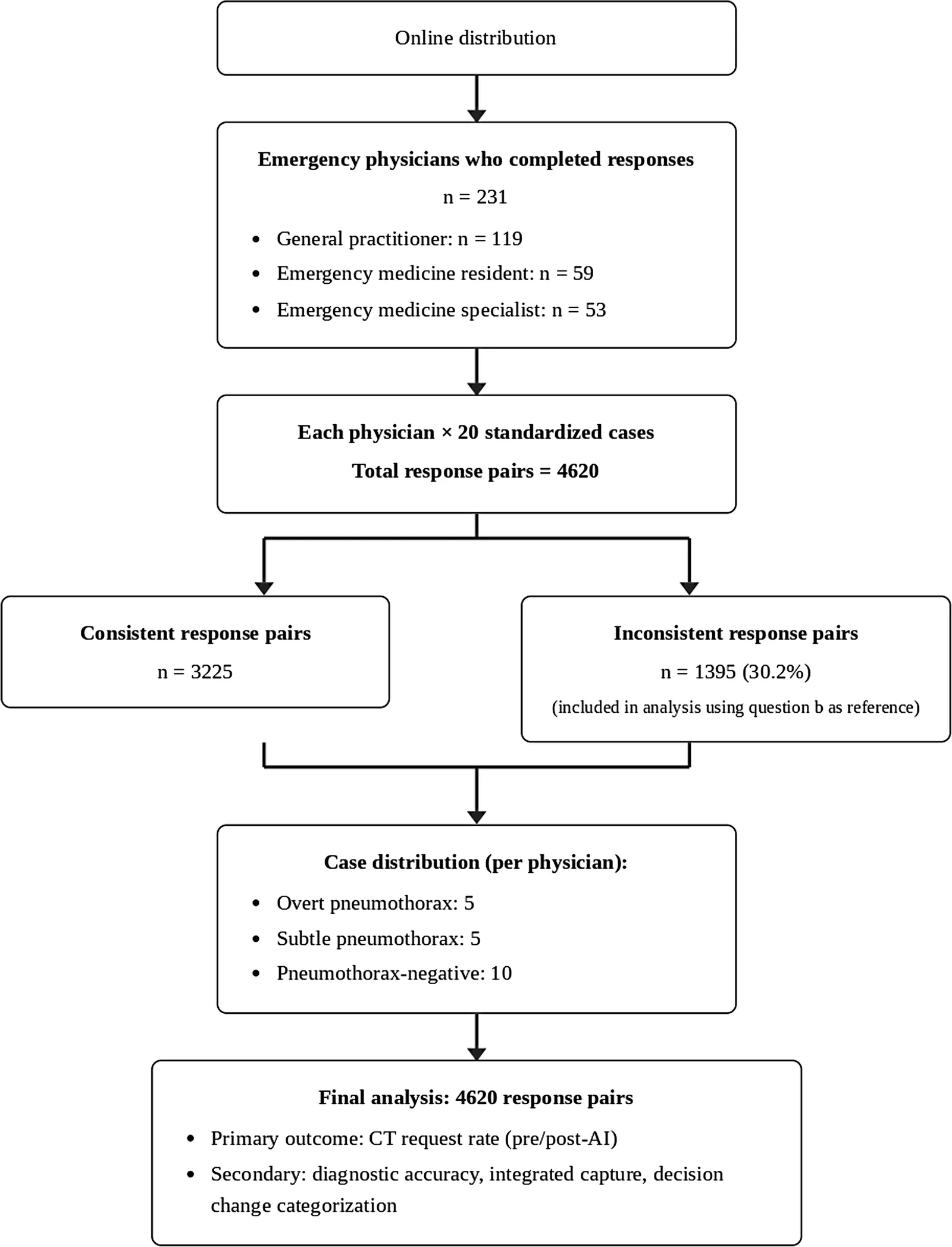
Participant and data flow diagram

The online survey was distributed between 1 and 15 April 2026, and 231 physicians completed the entire survey. Because the survey structure required every mandatory item to be answered, no participants were excluded for missing data. The final analysis comprised 4,620 physician–case response pairs derived from the 20 standardised cases evaluated by each of the 231 physicians. The participant flow is shown in Fig. 1. Slightly more than half of the participants were general practitioners (*n* = 119; 51.5%); emergency medicine residents accounted for 25.5% (*n* = 59) and emergency medicine specialists for 22.9% (*n* = 53). Within the seniority distribution, the largest group consisted of recent graduates (*n* = 99; 42.9%); physicians with 10 or more years of experience represented 16.9%. The full title and seniority distribution is shown in Table 1.

**Table 1:** Demographic characteristics of participating emergency physicians

Variable	Category	*n*	%
Professional title	General practitioner	119	51.5
	Emergency medicine resident	59	25.5
	Emergency medicine specialist	53	22.9
Seniority	Recent graduate	99	42.9
	1–3 years	41	17.7
	4–10 years	52	22.5
	10 + years	39	16.9
Total		231	100.0

Percentages were calculated over the total number of participants (*n* = 231)

Findings on the primary endpoint — the change in simulated CT-ordering intent — are presented below. Overall, simulated CT-ordering intent across all cases was 8.1% before AI-assisted interpretation (*n* = 372/4,620) and rose to 26.7% (*n* = 1,233/4,620) after the AI alert; the absolute change was + 18.6% points (McNemar exact test, *p* < 0.001).

The effect size differed markedly by case type. In pneumothorax-positive cases, simulated CT-ordering intent rose from 9.0% to 40.6% (Δ=+31.6 points; *p* < 0.001). In pneumothorax-negative cases the increase was limited: 7.1% → 12.8% (Δ=+5.7 points; *p* < 0.001). The effect was strongest in the subtle pneumothorax subgroup, where intent rose from 15.8% to 51.1% (Δ=+35.2 points; *p* < 0.001); in the overt pneumothorax subgroup the increase was from 2.2% to 30.0% (Δ=+27.9 points; *p* < 0.001). All case-type comparisons are presented in Table 2.

**Table 2:** Pre- and post-AI chest CT ordering rates by case type

Case group	*n*	Pre-AI CT *n* (%)	Post-AI CT *n* (%)	Δ (points)	*p*
All cases (overall)	4,620	372 (8.1)	1,233 (26.7)	+ 18.6	< 0.001
Pneumothorax-positive	2,310	208 (9.0)	937 (40.6)	+ 31.6	< 0.001
Pneumothorax-negative	2,310	164 (7.1)	296 (12.8)	+ 5.7	< 0.001
Overt pneumothorax	1,155	25 (2.2)	347 (30.0)	+ 27.9	< 0.001
Subtle pneumothorax	1,155	183 (15.8)	590 (51.1)	+ 35.2	< 0.001

*p* values were calculated using the McNemar exact test (two-sided). Δ: absolute percentage-point change (post − pre)

To examine the direction in which this increase occurred, the distribution of decision changes was analysed across four categories: “moved to CT” (did not order CT pre-AI, ordered CT post-AI), “dropped CT” (ordered CT pre-AI, did not order CT post-AI), “persisted with CT” (ordered CT in both conditions), and “already not ordering” (did not order CT in either condition). Overall, 966 of the 4,620 responses (20.9%) reflected a move towards CT and 105 (2.3%) a move away from CT; the net decision change was an increase of 861 responses.

Across case types, the “moved to CT” proportion was dominant in pneumothorax-positive cases (33.6%) and limited in pneumothorax-negative cases (8.2%). The highest “moved to CT” proportion was observed in the subtle pneumothorax subgroup (39.1%). The full distribution is shown in Table 3.

**Table 3:** Distribution of changes in CT ordering decision after the AI alert

Case group	*n*	Moved to CT *n* (%)	Dropped CT *n* (%)	Persisted with CT *n* (%)	Already not ordering *n* (%)
Overall	4,620	966 (20.9)	105 (2.3)	267 (5.8)	3,282 (71.0)
Pneumothorax-positive	2,310	777 (33.6)	48 (2.1)	160 (6.9)	1,325 (57.4)
Pneumothorax-negative	2,310	189 (8.2)	57 (2.5)	107 (4.6)	1,957 (84.7)
Overt pneumothorax	1,155	325 (28.1)	3 (0.3)	22 (1.9)	805 (69.7)
Subtle pneumothorax	1,155	452 (39.1)	45 (3.9)	138 (11.9)	520 (45.0)

Moved to CT = responses that shifted to a post-AI CT order; Dropped CT = responses that dropped the CT order after the AI alert

Interpreting the effect of the AI alert correctly requires knowledge of the physicians’ baseline diagnostic performance in the absence of assistance. On chest radiograph interpretation alone, the overall sensitivity for pneumothorax was 81.1%, specificity 91.3%, and overall accuracy 86.3%. When “not sure” responses were treated as misclassifications, the strict sensitivity dropped to 73.8% and the strict specificity to 84.8%.

Physician sensitivity was 94.9% in the overt pneumothorax subgroup and declined to 65.1% (strict sensitivity 54.8%) in the subtle pneumothorax subgroup. Specificity remained above 90% in all groups. By professional title, emergency medicine specialists showed the highest sensitivity (85.9%) and specificity (98.0%); the corresponding values were 78.6% and 91.0% in general practitioners and 81.8% and 86.0% in residents. The diagnostic performance values for all groups are shown in Table 4.

**Table 4:** Pneumothorax diagnostic performance of physicians without AI assistance

Group	Sens. (%)	Spec. (%)	Sens. strict (%)	Spec. strict (%)	Overall acc. (%)
Overall (all cases)	81.1	91.3	73.8	84.8	86.3
Overt pneumothorax	94.9	91.3	92.8	84.8	92.6
Subtle pneumothorax	65.1	91.3	54.8	84.8	83.2
General practitioner	78.6	91.0	70.8	85.4	84.9
Emergency medicine resident	81.8	86.0	73.7	79.0	83.9
Emergency medicine specialist	85.9	98.0	80.6	90.2	91.9
Recent graduate	76.5	90.2	68.3	83.2	83.4
1–3 years	77.4	83.4	68.5	74.9	80.4
4–10 years	90.9	93.3	85.0	88.5	92.1
10 + years	82.9	99.5	78.5	94.6	91.2

Sens. = sensitivity; Spec. = specificity; Overall acc. = overall accuracy. In the base calculation, “not sure” responses were excluded from the analysis; in the strict versions, those responses were considered as misclassifications

The combined effect of the physician’s baseline assessment and the post-AI decision constitutes the clinically most important outcome of the study. In pneumothorax-positive cases, the rate at which physicians answered “PTX present” without AI assistance was 73.8% (*n* = 1,705/2,310); when the integrated capture definition (the physician’s baseline “PTX present” answer or a post-AI CT order) was applied, this rate rose to 91.0% (*n* = 2,101/2,310; net improvement + 17.1 points).

In the subtle pneumothorax subgroup the improvement was + 30.2 points (54.8% → 85.0%); in the overt pneumothorax subgroup it was + 4.1 points (92.8% → 96.9%). Among the 339 responses in which physicians had initially answered “PTX absent” in the subtle pneumothorax subgroup, 211 (62.2%) were converted into a CT order after the AI alert.

The improvement in integrated capture rates was of comparable magnitude across professional titles: +29.7 points in general practitioners, + 31.9 points in emergency medicine residents, and + 29.4 points in emergency medicine specialists. All values are presented in Table 5.

**Table 5:** Integrated diagnostic capture rate after the AI alert

Group	*n*	Pre-AI “PTX present” *n* (%)	Post-AI integrated capture *n* (%)	Net improvement (points)
All pneumothorax-positive cases	2,310	1,705 (73.8)	2,101 (91.0)	+ 17.1
Overt pneumothorax	1,155	1,072 (92.8)	1,119 (96.9)	+ 4.1
Subtle pneumothorax	1,155	633 (54.8)	982 (85.0)	+ 30.2
General practitioner (subtle)	595	297 (49.9)	474 (79.7)	+ 29.7
Emergency medicine resident (subtle)	295	170 (57.6)	264 (89.5)	+ 31.9
Emergency medicine specialist (subtle)	265	166 (62.6)	244 (92.1)	+ 29.4

Integrated capture: the proportion of pneumothorax-positive cases in which the physician either answered “PTX present” at baseline or ordered a chest CT after the AI alert

How the overall effect size is distributed across physician groups is an important question for the practical deployment of the system. The increase in simulated CT-ordering intent was statistically significant in every title stratum. The largest absolute increase was observed among general practitioners (+ 22.2 points; 8.0% → 30.2%; *p* < 0.001). The increase was + 12.8 points in emergency medicine residents (9.0% → 21.8%; *p* < 0.001) and + 17.2 points in emergency medicine specialists (7.1% → 24.2%; *p* < 0.001). The corresponding net decision-change counts were 676, 171, and 224 responses, respectively (Table 6).

**Table 6:** Pre- and post-AI chest CT ordering rates by professional title

Title	*n*	Pre-AI CT *n* (%)	Post-AI CT *n* (%)	Δ (points)	*p*	Net changed *n*
General practitioner	2,380	191 (8.0)	719 (30.2)	+ 22.2	< 0.001	676
Emergency medicine resident	1,180	106 (9.0)	257 (21.8)	+ 12.8	< 0.001	171
Emergency medicine specialist	1,060	75 (7.1)	257 (24.2)	+ 17.2	< 0.001	224

Net changed = sum of responses that shifted towards CT and away from CT after AI exposure

Along the seniority axis, the largest increase was observed in the recent graduate group (+ 25.3 points; 9.2% → 34.4%; *p* < 0.001). The absolute changes in the 1–3 years, 4–10 years, and 10 + years groups were + 14.3, + 12.8, and + 14.2 points, respectively; *p* < 0.001 in all groups (Table 7).

**Table 7:** Pre- and post-AI chest CT ordering rates by seniority

Seniority	*n*	Pre-AI CT *n* (%)	Post-AI CT *n* (%)	Δ (points)	*p*	Net changed *n*
Recent graduate	1,980	182 (9.2)	682 (34.4)	+ 25.3	< 0.001	620
1–3 years	820	89 (10.9)	206 (25.1)	+ 14.3	< 0.001	145
4–10 years	1,040	61 (5.9)	194 (18.7)	+ 12.8	< 0.001	179
10 + years	780	40 (5.1)	151 (19.4)	+ 14.2	< 0.001	127

In the title × case-type cross-analysis, emergency medicine specialists showed the largest increase in subtle pneumothorax cases (+ 42.6 points; 12.5% → 55.1%; *p* < 0.001). In general practitioners the increases were similar between overt (+ 36.6 points) and subtle (+ 36.1 points) cases. In pneumothorax-negative cases the increase was limited across all title strata (between + 3.2 and + 8.0 points). All values from the cross-analysis are presented in Table 8.

**Table 8:** Professional title × case type cross-analysis

Title × Case type	*n*	Pre-AI CT *n* (%)	Post-AI CT *n* (%)	Δ (points)	*p*
General practitioner — Overt PTX	595	15 (2.5)	233 (39.2)	+ 36.6	< 0.001
General practitioner — Subtle PTX	595	102 (17.1)	317 (53.3)	+ 36.1	< 0.001
General practitioner — No PTX	1,190	74 (6.2)	169 (14.2)	+ 8.0	< 0.001
Emergency medicine resident — Overt PTX	295	10 (3.4)	63 (21.4)	+ 18.0	< 0.001
Emergency medicine resident — Subtle PTX	295	48 (16.3)	127 (43.1)	+ 26.8	< 0.001
Emergency medicine resident — No PTX	590	48 (8.1)	67 (11.4)	+ 3.2	< 0.001
Emergency medicine specialist — Overt PTX	265	0 (0.0)	51 (19.2)	+ 19.2	< 0.001
Emergency medicine specialist — Subtle PTX	265	33 (12.5)	146 (55.1)	+ 42.6	< 0.001
Emergency medicine specialist — No PTX	530	42 (7.9)	60 (11.3)	+ 3.4	0.022

Physicians’ overall perceptions of the system were evaluated through the final survey items. For the question “Can AI-assisted chest radiograph interpretation reduce unnecessary chest CT use in the emergency department?” responses on the 1–5 scale yielded a mean of 1.91 ± 0.68 (median 2; IQR 1–2); 186 of the 231 physicians (80.5%) answered “definitely yes” or “yes.” For “the usability of such an AI support system in daily clinical practice,” responses on the 1–10 scale yielded a mean of 8.09 ± 1.52 (median 8; IQR 7–10); 193 physicians (83.5%) gave a score of 7 or higher. All values are shown in Table 9.

**Table 9:** Physicians’ perceptions of the AI support system

Item	Mean ± SD	Median [IQR]	Positive view *n* (%)	Negative/neutral *n* (%)
Can AI reduce unnecessary chest CT? (1–5)	1.91 ± 0.68	2 [1–2]	186 (80.5)	45 (19.5)
Usability of the AI support system (1–10)	8.09 ± 1.52	8 [7–10]	193 (83.5)	38 (16.5)

Positive-view thresholds: score ≤ 2 for Item 1 (“definitely yes” or “yes” on the 1–5 scale); score ≥ 7 for Item 2 (high usability on the 1–10 scale)

Finally, the pattern of inconsistent responses arising as a natural consequence of the survey design was examined. In 1,395 of the 4,620 response pairs (30.2%) a logical inconsistency was identified between questions a and b (e.g. a physician answering “PTX present” at baseline but selecting “The AI made me drop the CT” after AI exposure). These responses were handled as described in the Methods, with question b taken as the reference in the CT ordering analysis because it reflects the physician’s final stance. The inconsistency rate differed markedly by case type: 867/2,310 (37.5%) in pneumothorax-positive cases and 528/2,310 (22.9%) in pneumothorax-negative cases. To assess the robustness of the primary endpoint, we conducted a pre-specified sensitivity analysis excluding these 1,395 inconsistent pairs and interpreted it alongside the full-data analysis. In the inconsistency-excluded analysis, the direction and significance of the primary endpoint were preserved: simulated CT-ordering intent rose from 11.5% to 16.7% (Δ=+5.1 points; *p* < 0.001), although the absolute effect size was smaller than in the full-data analysis (8.1% to 26.7%). In subgroup analyses, the increase remained significant in subtle pneumothorax cases (Δ=+18.6 points; *p* < 0.001) but was no longer significant in pneumothorax-negative cases (Δ=+0.3 points; *p* = 0.71). This pattern indicates that the principal finding, the concentration of increased simulated CT-ordering intent in true positive cases, is robust to the handling of inconsistent responses.

## Discussion

The principal finding of this study is that AI-assisted chest radiograph interpretation significantly modifies the pneumothorax diagnostic decisions and chest CT ordering behaviour of emergency physicians, but does so in a direction opposite to the one initially anticipated. Simulated CT-ordering intent rose from 8.1% to 26.7% after the AI alert; however, the bulk of this increase occurred in true positive cases, and the rise remained quite limited in pneumothorax-negative cases. In subtle pneumothorax cases, the integrated diagnostic capture rate rose from 54.8% to 85.0%; among the cases in which physicians had initially answered “no pneumothorax,” 62.2% were converted into a CT order after the AI alert. This pattern indicates that the AI system functions in the emergency department less as a stand-alone diagnostic tool and more as a second reader that captures findings overlooked by the physician.

Explaining this pattern requires focusing on the study’s hypothesis-opposite finding. The study was originally designed under the hypothesis that AI support would reduce unnecessary chest CT ordering. The findings run counter to that expectation. Three complementary explanations are relevant.

First, the case mix was demanding by design. Half of the pneumothorax-positive pool consisted of subtle cases, and the entire pneumothorax-negative pool consisted of symptomatic patients (chest pain, cough) that legitimately raise clinical suspicion. The fact that the AI alert generated additional imaging requests in such a reader pool — confronted with difficult cases rather than the routine flow of an emergency department — is a natural outcome.

Second, physicians tend to interpret a positive AI annotation as an alert for a “must-not-miss pathology.” This pattern, defined in the literature as AI-induced confirmation seeking, denotes the clinician’s tendency to seek further imaging to verify a finding flagged by AI [14, 16]. The behaviour is clinically rational: in a case where the AI algorithm might be a false positive, confirming the finding with CT is a defensible decision from a patient safety standpoint.

Third, the four options offered in question b did not contain a clear field corresponding to “the AI made me decide to order a CT.” As a consequence, physicians who did not initially intend to order a CT but changed their minds after the AI alert may have distributed their answers between “I want a CT despite the AI” and “I was going to anyway and still would.” This survey-instrument limitation must be borne in mind when interpreting the absolute CT increase; importantly, however, it does not undermine the study’s principal finding — the marked improvement in the integrated capture rate.

The terrain on which these explanations rest aligns with the chain of evidence from recent MRMC and simulation-based studies. Across emergency-physician and mixed-reader cohorts — from a 200-physician study by the Macquarie group, where the effect was not always directed correctly [15], to sequential simulation and multidisciplinary MRMC work [4, 5] and a three-centre cohort in which inter-observer AUC rose from 0.861 to 0.886 [17] — AI support has consistently improved diagnostic performance, with stronger effects among less experienced readers. The shared message is that AI functions as a “second reader” in chest radiograph interpretation; our findings extend that thesis into emergency-department practice and the CT-ordering decision.

Comparable national and regional data reinforce this: a Türkiye model trained on a 152-radiograph cohort outperformed standalone physician performance across 15 specialties, reaching 0.93 sensitivity in subtle pneumothorax versus 0.55–0.84 for physicians [8], and a Taiwanese study reported a standalone AI AUC of 0.965 with significant improvement across all reader groups [7]. Our study adds two elements: it is the first large-sample Türkiye study to measure an immediate behavioural endpoint (the CT-ordering decision), and the first pneumothorax-specific assessment of an MDR-CE certified tool already in routine emergency-department use.

Within this literature framework, the clinically strongest message of our study is that the principal contribution of AI assistance emerges in subtle cases. While physician sensitivity in the overt pneumothorax subgroup is already 92.8%, in the subtle subgroup it falls to 54.8%. After the AI alert, the integrated capture rate in this challenging subgroup rose to 85.0%, and 62.2% of the responses in which physicians had answered “no pneumothorax” were converted into a CT order.

This pattern is consistent with the existing literature. In Plesner et al.‘s four-centre evaluation of commercial AI tools, sensitivity for small pneumothoraces ranged from as low as 9% to above 85% across tools [9], and in a follow-up AI-assisted study of the same Munich 563-radiograph cohort cited above [3], the pneumothorax AUROC of non-radiologist trainees rose from 0.846 to 0.974 with AI support, corresponding to an absolute sensitivity gain of approximately 30% in these cases [6]. Together these confirm that subtle pneumothorax is a systematic source of missed findings for inexperienced readers and that AI support is well suited to fill this gap. Our data extend the argument: not only diagnostic accuracy but also concrete decision-level behaviour (the CT order) shifts in the right direction with the AI alert.

AI support also affects physician groups unequally. In the seniority analysis, the largest behavioural change was observed in the recent graduate group (+ 25.3 points); in the 1–3 years, 4–10 years, and 10 + years groups the effect ranged between + 12.8 and + 14.3 points. This pattern indicates that AI support can produce a larger behavioural effect in less experienced physicians, but two caveats apply.

First, gain and risk concentrate in the same group. The Taiwanese MRMC study found the largest relative AUC improvement in the most junior readers (a negative, significant AI × seniority interaction) [7], yet the Cologne mammography study showed that inexperienced readers are also the most susceptible to incorrect AI suggestions [18]. The AI effect in junior physicians is therefore bidirectional: a correct alert helps most, but an incorrect one can harm most. AI should reach this group within a framework of training and oversight.

Second, the large increase observed in emergency medicine specialists in subtle cases in our study (+ 42.6 points) indicates that experience is not a “sufficient safeguard.” This finding suggests that AI assistance may add meaningful value not only for trainees but also for experienced physicians.

The literature on decision support and CT ordering is itself divergent. In the five-centre EmbED study, an EHR-integrated tool reduced CT use by 6–10% among high-ordering physicians while slightly increasing it among low-ordering ones [12], whereas a Calgary cluster-randomised trial found no significant effect (OR 0.93; *p* = 0.31) [13]. Reviews of machine-learning decision support conclude that any effect is strongly conditioned by the system, context, and manner of deployment, and that the evidence on whether such tools improve clinician performance remains methodologically limited [14, 16].

Within this literature, our findings occupy a distinct position. In this simulation, AI annotation was associated with an increase in simulated CT-ordering intent that was concentrated in CT-confirmed (true positive) cases and limited in negative cases. The fact that the increase in pneumothorax-negative cases was limited to only + 5.7 points (compared with + 31.6 points in pneumothorax-positive cases) supports the conclusion that the AI alert does not generate false-positive redirection. We interpret this as a pattern consistent with more selective imaging intent rather than as evidence of improved real-world CT appropriateness, which our design cannot establish. The study did not evaluate actual CT ordering, downstream clinical management, emergency department length of stay, missed-diagnosis rates, adverse events, or patient outcomes.

We now consider the response inconsistencies within the framework of automation bias, one of the most important risks in AI-assisted decision-making, whereby the physician accepts a wrong AI alert without questioning (commission error) or misses a pathology the AI did not flag (omission error). The literature shows that reliance on automation grows as perceived system reliability, time pressure, and cognitive load increase, with verification behaviour declining and error rates paradoxically rising [19, 20]; notably, simply labelling a diagnostic suggestion as AI-generated lowered physicians’ accuracy when the suggestion was incorrect, even when it had in fact been produced by expert physicians [21].

In our study, a logical inconsistency between questions a and b was identified in 1,395 of the 4,620 response pairs (30.2%). At first glance this rate is high, but it becomes interpretable in light of the literature. Some inconsistencies may reflect appropriate reliance, the physician revising the baseline decision after the AI alert in the direction of the patient’s true diagnosis; others may reflect automation bias, an originally correct baseline decision being changed after an incorrect AI output. The inconsistency rate in pneumothorax-positive cases (37.5%) was markedly higher than in negative cases (22.9%): when the physician had answered “PTX absent” at baseline, the AI’s positive annotation more often triggered a decision change. Although this does not in itself prove bias, it indicates that the line between appropriate reliance and automation bias could be measured more cleanly in future studies through a redesigned set of options for question b.

Considered together, our findings carry three implications for emergency-department practice. First, AI support offers a meaningful diagnostic safety layer where 24/7 radiologist access is limited — a structural reality in many countries, Türkiye included; the 30.2-point gain in integrated capture for subtle pneumothorax represents patients who might otherwise be missed on night shifts or when radiology consultation is delayed.

Second, AI assistance should not be positioned as a simple “CT-reduction tool.” Our data show that the AI alert directs physicians towards CT ordering in true positive cases while adding only limited ordering in true negative cases, clarifying how the trade-off between diagnostic gain (capturing missed findings) and cost (unnecessary CT) plays out in practice. This positioning extends beyond imaging: in a vision-enabled GPT study on spontaneous pneumothorax from the same country, the model was framed as adjunct decision support within a human-in-the-loop workflow with mandatory clinician review, not as an autonomous tool [10] — a convergent message reinforcing that the value of AI lies in assisting, not replacing, the physician’s final decision.

Third, the finding that AI support produces a significant effect across all experience levels — from recent graduates to senior specialists — indicates that the system can be positioned as a shared additional decision layer for the entire emergency department team. Such deployment, however, must be planned within a framework of automation bias training and clinical oversight.

The principal strength is the sample size: 231 physicians and 4,620 physician–case response pairs exceeds that of many comparably designed MRMC and simulation studies [5–7, 15]. Other strengths include a chest-CT gold-standard reference for every case, which minimises misclassification; randomised case order, which mitigated fatigue and learning effects; the use of an MDR-CE Class IIa certified tool in routine clinical use rather than a research prototype; and nationwide distribution yielding broad title and seniority diversity.

The study also has several limitations, and the findings should be interpreted accordingly. Three features in particular constrain the generalisability of this work and should be borne in mind throughout: the study was conducted at a single centre, it used an online survey-based simulation rather than real-world clinical workflow, and it evaluated a small, fixed set of 20 radiographs; each is elaborated below. First, the study is based on a simulation survey design. Real-world clinical decision-making emerges from the combination of detailed patient history, physical examination, ancillary laboratory tests, the physician’s real-time cognitive load, and the patient–physician interaction. The survey environment necessarily simplified most of these elements; each case was accompanied only by a brief clinical context label consisting of age and main symptoms.

Second, the pre- and post-AI items did not capture CT-ordering intent in an identical way. At baseline, only an “I am not sure, I would order a CT” response was coded as a CT order, so a physician who answered “pneumothorax present” was coded as not ordering CT even though such a physician might in practice proceed to CT, consultation, or another management pathway. This asymmetry means the endpoint reflects simulated imaging intent under the predefined coding scheme rather than directly comparable CT-ordering behaviour, and it is the central interpretive limitation of the study. Relatedly, the response options for question b did not contain a clear field corresponding to “the AI made me decide to order a CT,” which complicates interpretation of the absolute increase, although the integrated capture analysis partially mitigates it.

Third, the identification of a logical inconsistency in 30.2% of the 4,620 responses reflects an internal-consistency weakness of the survey instrument. As specified in the Methods, these responses were retained in the analysis by treating question b as the final stance, and this aspect of the survey design should be reinforced in future studies. As reported in the Results, a pre-specified sensitivity analysis excluding the inconsistent responses preserved the direction and significance of the primary endpoint, supporting the robustness of the principal finding.

Fourth, the 20 cases were drawn from the PACS archive of a single centre (Mardin Training and Research Hospital). This may introduce a limitation in case diversity; multicentre case pools drawn from different geographic regions and patient populations could yield more generalisable results.

Fifth, the overt/subtle pneumothorax distinction was made through a subjective classification based on visual volume assessment (approximately a 50% involvement threshold); formal volumetric methods such as the Light index or Collins measurement were not used. This may have introduced some classification bias into the subgroup comparisons.

Sixth, because the survey was distributed online and participation was voluntary, participant selection was non-probabilistic (convenience sampling). Physicians with an interest in artificial intelligence technologies or with a higher propensity to participate in online research may be over-represented; this somewhat restricts the generalisability of the findings.

Seventh, the study evaluated only one AI tool; different algorithms may produce different results in the same scenario. In Plesner et al.‘s comparison of four commercial tools, sensitivity for small pneumothorax ranged from 9% to 86% across tools [9]; our findings therefore cannot be directly extended to all commercial systems.

Eighth, although the AI tool used was focused on eight pathologies, the real-world spectrum of chest radiograph interpretation extends well beyond those bounds. López Alcolea et al.‘s emergency department cohort study showed that finding categories not covered by a commercial AI tool occur with non-trivial prevalence: approximately 16% mediastinal pathologies, 20% surgical material, and 20% other pulmonary findings (particularly hyperinflation) [22]. This indicates that AI-assisted interpretation should sit alongside, not replace, physician evaluation; in our study as well, the AI alert was positioned not as the sole determinant of the clinical decision but as a decision input preceding the physician’s final word.

Ninth, the study measured at a single point in time. With long-term use, physicians’ patterns of interaction with the AI system — developing automation bias or, conversely, algorithm rejection — may change. Such patterns can only be revealed by prospective follow-up studies.

Tenth, although the reference diagnosis for all cases was verified by chest CT, the study design did not track individual patient outcomes (e.g. length of treatment, admission rate, or mortality). Whether AI-assisted decision changes translate into actual patient outcomes remains the subject of prospective clinical impact studies. Finally, because the image set contained only CT-confirmed cases with concordant AI output, no AI false-positive or false-negative outputs were present; our findings therefore characterise physician responses to correct AI output and cannot speak to automation bias under realistic AI error conditions. A comparable confirmed-case design and the same transparent limitation have been adopted in related emergency-department pneumothorax work [10]. Evaluating behaviour under realistic AI errors requires a dedicated design incorporating curated AI error cases and is an important direction for future work.

## Conclusion

This study shows that AI-assisted chest radiograph interpretation significantly alters the pneumothorax diagnostic decisions and chest CT ordering behaviour of emergency physicians. The simulated CT-ordering intent did not decrease but rather increased; that increase, however, was concentrated in true positive cases and remained limited in pneumothorax-negative cases. The fact that approximately two-thirds of the pneumothorax diagnoses initially missed by physicians in subtle cases were converted into a CT order after the AI alert demonstrates that the system should be positioned not as a simple “CT-reduction tool” but as a second reader that captures findings the physician has overlooked. Prospective clinical impact studies are needed to determine how these findings translate into actual patient outcomes.

## Supplementary Information

Below is the link to the electronic supplementary material.

Supplementary Material 1

## Data Availability

The datasets used and/or analysed during the current study are available from the corresponding author on reasonable request.

## Declarations

### Use of AI tools in manuscript preparation

During the preparation of this manuscript, the authors used Claude (Anthropic, San Francisco, CA, USA), a large language model, to assist with manuscript layout, table visualisation, and reference formatting. All scientific content, data interpretation, statistical analyses, and conclusions are the authors’ own work. The authors reviewed and verified all outputs and assume full responsibility for the accuracy and integrity of the final manuscript.
