# Supplementary material for: Artificial intelligence as a second reader for pneumothorax detection: redirecting rather than reducing chest CT use
Source: BMC Emerg Med. 2026 Jul 2;26:256. doi: 10.1186/s12873-026-01673-x (PMC13599224; doi:10.1186/s12873-026-01673-x)
Supplement: Supplementary file 1 [file 12873_2026_1673_MOESM1_ESM.docx]

**Table S1. Sensitivity analysis excluding inconsistent response pairs (n=1,395)**

| **Case group** | **n** | **Pre-AI CT n (%)** | **Post-AI CT n (%)** | **Δ (points)** | **p** |
| --- | --- | --- | --- | --- | --- |
| All cases (overall) | 3,225 | 372 (11.5) | 537 (16.7) | +5.1 | <0.001 |
| Pneumothorax-positive | 1,443 | 208 (14.4) | 368 (25.5) | +11.1 | <0.001 |
| Pneumothorax-negative | 1,782 | 164 (9.2) | 169 (9.5) | +0.3 | 0.71 |
| Overt pneumothorax | 700 | 25 (3.6) | 47 (6.7) | +3.1 | <0.001 |
| Subtle pneumothorax | 743 | 183 (24.6) | 321 (43.2) | +18.6 | <0.001 |

*Inconsistent response pairs (n=1,395) were excluded from the analysis. p values were calculated using the McNemar exact test (two-sided). Δ: absolute percentage-point change (post − pre). The loss of statistical significance in pneumothorax-negative cases indicates that the AI alert did not redirect physician decisions in true negative cases.*
